# Seed Germination in *Phragmites australis* and *P. mauritianus*: Effects of Salinity and Thermoperiod

**DOI:** 10.1002/pei3.70091

**Published:** 2025-10-06

**Authors:** L. P. Tshapa, K. K. Naidoo, S. Shaik, G. Naidoo

**Affiliations:** ^1^ School of Life Sciences University of KwaZulu‐Natal Pietermaritzburg Westville South Africa; ^2^ Department of Nature Conservation Mangosuthu University of Technology Jacobs South Africa

**Keywords:** climate change, reeds, salt tolerance, seed vigor, thermoperiods, viability

## Abstract

Understanding species‐specific salt and heat tolerance mechanisms provides valuable insights into colonization and zonation patterns in saline environments. To explore these mechanisms, this study investigated the effects of selected salinity and thermoperiod on seed germination in the African haplotypes of the common reeds, *P. australis* and *P. mauritianus* . The effect of salinity was determined by germinating seeds in 0%, 5%, 10%, 20%, and 50% seawater at alternating night/day temperatures of 15°C/25°C and 20°C/30°C for 21 days. In both species, the highest germination, seedling vigor, root length, and number of leaves were obtained in the non‐saline control treatment. In *P. australis,* there was 100% seed germination in the non‐saline controls in both thermoperiods, while in *P. mauritianus* , germination was 36% and 45% lower, respectively. Salinity did not affect the germination of *P. australis* at 15°C/25°C, but at 20°C/30°C, germination decreased. In *P. mauritianus* , seed germination decreased significantly with an increase in salinity in both thermoperiods. Salinity and 20°C/30°C thermoperiod significantly reduced biomass, leaf production, culm height, and root elongation in both species. *Phragmites australis* was more salt‐tolerant than *P. mauritianus* , as germination percentage, biomass, root length, and seedling vigor index were higher in both thermoperiods. Neither species germinated at 5°C and 35°C/40°C thermoperiods; however, *P. australis* seeds exhibited higher viability as indicated by a greater germination recovery percentage compared to *P. mauritianus* . *Phragmites australis* seeds are lighter, fluffier, more viable, disperse easily, and may contribute to its ability to colonize a greater diversity of habitats compared to *P. mauritianus* .

## 1 Introduction

The common reed, *Phragmites australis* (Cav.) Trin. ex Steud. (Poaceae) is a widespread, highly productive, perennial species in fresh and brackish wetlands along rivers and estuaries. Its worldwide distribution is attributed to its high genetic diversity and several ploidy levels, which contribute to its phenotypic plasticity (Eller and Brix 2012; Meng et al. 2016). In many parts of the world, *Phragmites* stands are dense, monospecific, invasive, and tend to reduce biodiversity and species composition (Kettenring et al. 2011, 2015; Fernandes and Adams 2016). There are four species of *Phragmites*: *P. australis* , *P. communis* , *P. karka* , and *P. mauritianus*. The two species of *Phragmites* in southern Africa are *P. australis* and *P. mauritianus* Kunth (Gordon‐Gray and Ward 1971; Naidoo 2021), and they are African haplotypes (haplotype K for *P. australis* and haplotype V and AP for *P. mauritianus* ) (Canavan et al. 2018).

*Phragmites australis* is an aggressive competitor that alters species composition and hydrological regimes (Achenbac et al. 2012; Catford and Jansson 2014). It is one of the first to colonize disturbed or reclaimed wetlands. Despite its invasive nature, *P. australis* provides many ecosystem services (Mmopelwa 2006; Kobbing et al. 2016), such as use in constructed wetlands for wastewater treatment. Indigenous people in South Africa use the plant for buildings, roofing material, and weaving mats. In addition, *Phragmites australis* has an extensive root system that minimizes erosion and maintains wetland integrity. While *P. australis* has been extensively studied in terms of its ecophysiological responses to stressors, little is known about *P. mauritianus* and its responses to environmental stressors. Native populations of *P. australis* and *P. mauritianus* are expanding in wetlands and disturbed areas in southern Africa (Fernandes and Adams 2016). Hybridization of these two closely related species could potentially enhance invasiveness and complicate control measures (Paul et al. 2010).

Field and genetic data suggested that both sexual and vegetative reproduction modes contribute to the establishment of new stands of *Phragmites* (Belzile et al. 2010). High genetic diversity among reed stands indicates the importance of sexual reproduction for dispersal (Chen et al. 2024). However, accidental transport of fragments through floods can also be a source of new individuals. Once initiated from seedlings or fragments, expansion occurs rapidly through vegetative growth (Hurley et al. 2024). Species using both vegetative propagation and sexual reproduction strategies, like *P. australis* , have higher invasion success than those using a single mode (Galatowitsch et al. 2016). Some studies suggest that the less dominant wetland species with a low seedling survival rate could be due to dormancy and the environmental requirements for germination (Baskin and Baskin 2003; Bischoff et al. 2006; Geissler and Gzik 2010). In addition, a similar reed, *Arundo donax* , primarily reproduces vegetatively, with sexual reproduction being rare or absent due to its triploid chromosome structure, which limits genetic diversity (Bucci et al. 2013). Therefore, understanding species‐specific germination requirements in relation to temperature, salinity, light, and soil moisture is essential for predicting plant colonization and invasion (Greenwood and MacFarlane 2006; Baskin and Baskin 2022).

Climate change, characterized by rising temperatures, altered precipitation patterns, increased salinity, and elevated atmospheric CO_2_ concentrations, poses substantial challenges to the germination of halophytes (Souid et al. 2016). Shifts in the timing and intensity of temperature and rainfall can disrupt germination cues, often resulting in poor germination success, seedling mortality, or failure to establish (Parihar et al. 2015). Elevated CO_2_ levels may further influence seed development by altering seed quality and dormancy regulation (Song et al. 2008). Germination in halophytes is highly temperature‐dependent, with each species exhibiting an optimal thermal range for successful germination (Seal and Dantas 2021). When environmental temperatures exceed these limits, germination may be inhibited due to thermal stress and impaired enzymatic activity (Lombardi and Bedini 2021). Temperature is particularly vital for germination success, as it influences the geographic and ecological distribution of *Phragmites* ecotypes (Greenwood and MacFarlane 2006; Eller et al. 2017). *Phragmites* species produce large quantities of small, wind‐pollinated seeds for establishing populations over large distances and vegetative structures, such as rhizomes and stolons, for locally expanding populations (Catford and Jansson 2014). *Phragmites communis* tolerates a wide temperature range; however, extreme heat and cold conditions, including frost, significantly reduce germination rates (Tougas‐Tellier et al. 2015; Oh et al. 2017). Optimal germination occurs between 16°C and 25°C, with diurnal temperature fluctuations further enhancing the process (Packer et al. 2017; Hurley et al. 2024). Whilst *P. australis* exhibits a broad temperature tolerance for germination, with optimal conditions between 10°C and 30°C, excessive salinity significantly hinders the germination speed and success of *P. australis* (Gorai et al. 2006; Roberts et al. 2023). In contrast, *P. communis* demonstrates remarkable resilience, maintaining germination even under hypersaline conditions up to 400 mM, highlighting the plasticity in adapting to salinity stresses. This adaptability arises from a combination of factors, including genotypic and phenotypic variations among seedlings, genetic diversity across populations, and ecological distribution, which influence seed viability, vigor, and seed‐setting capacity (Engloner 2009; Roy et al. 2024). Adaptive strategies for survival in hypersaline environments include seed dormancy and reduced seed viability, enabling halophytes to withstand challenging conditions (Lombardi and Bedini 2021). Notably, *P. karka* exhibits exceptional salt tolerance, with seeds capable of germinating in NaCl concentrations as high as 500 mM, facilitating its thriving in saline habitats (Zehra and Khan 2007).

Little to no information is available on seed germination of southern African populations, especially on *P. mauritianus*, and their responses to environmental stressors. *Phragmites australis* exhibits a cosmopolitan distribution, occurring globally across a wide range of habitats, while *P. mauritianus* has a more restricted distribution, primarily confined to specific regions (Pagad et al. 2022). Knowledge of the germination responses of these two coexisting reed species is essential, as the ecophysiological differences between genetic lineages facilitate invasion success (Mozdzer and Zieman 2010). Seed germination behavior in relation to thermal and salinity stress is essential to determine the colonization capacity of these species in southern African wetlands. In this study, we determined the effect of selected thermoperiods and salinity on seed germination of *P. australis* and *P. mauritianus* . We hypothesized that *P. australis* seeds are viable and exhibit greater tolerance to salinity compared to *P. mauritianus* , owing to their wide distribution and an enhanced capacity to acclimate to environmental stressors. The treatment levels in this study were chosen within the ranges encountered in South African wetlands where these species co‐occur. Understanding how these species respond to stressors will help predict their distribution patterns in response to sea‐level rise, climate change, and overall vigor.

## 2 Materials and Methods

### 2.1 Germination Experiments

Inflorescences containing mature seeds of *P. australis* and *P. mauritianus* were collected during the winter (June 2023 and 2024) from the fields in Isipingo, Durban (29.97301° S, 30.96066° E), and Umlazi (29.57342° S, 30.56410° E). The mean annual temperature in this area was 20°C, and the mean annual rainfall was 1061 mm in 2023 (Climate‐Data.org 2025). A key morphological distinction between selected species lies in their leaf‐shedding patterns: in *P. mauritianus* , both the leaf blade and sheath are shed together, exposing the internodes and axillary buds, whereas in *P. australis* , only the blade is deciduous, and the sheath remains attached to the stem (Naidoo 2021). Fresh seeds were sterilized using 0.85% Clorox bleach for 1 min prior to the commencement of germination experiments. Images of the seeds were captured using a Nikon Eclipse AZ100 microscope (Nikon, Japan). Seed weight was measured using a precision balance (SigmaAldrich, USA) based on 150 seeds (*n* = 5) (see S2 in the supplementary data). The seeds were placed on two layers of Whatman No. 1 filter paper (90 mm) inside a 90 mm tight‐fitting Petri dish. The filter paper was moistened with either 7 mL of seawater treatments or distilled water. Seawater was collected from uShaka Marine World (Durban, South Africa).

The effects of salinity and thermoperiod were determined by germinating seeds in solutions of 0%, 5%, 10%, 20%, and 50% seawater at alternating day/night thermoperiods of 15°C/25°C and 20°C/30°C, with a 16‐h day and 8‐h night cycle. These seawater concentrations are equivalent to 0, 25, 50, 100, and 250 mM NaCl (Munns et al. 2006). Seawater was diluted with distilled water to achieve the desired concentrations, and salinity was measured using a Bante multimeter (Bante instrument, China) to ensure accuracy. The treatment levels in this study were chosen within the temperature ranges encountered in South African wetlands where these species co‐occur. Using natural seawater for accurate salinity stress assessments was important, especially when trying to emulate the effect of sea‐level rise. Petri dishes were sealed with parafilm to reduce evaporation. There were five replicates of 25 seeds of each species per Petri dish. Petri dishes were placed in a growth chamber (Jeiotech, Korea), and germination was monitored daily for 21 days. Germination was defined as the emergence of a radicle greater than 1 mm. The number of days taken to achieve 50% of the final germination capacity was used to compare germination rates at the different salinities, and germination speed (S) was calculated using the formula below (Chiapusio et al. 1997).

$$S=\left(G_{1}+G_{2}+\ldots +G_{n}\right)/\left(T_{1}+T_{2}+\ldots +T_{n}\right)$$

*G*_*n*_, number of seeds germinated on day *n*; *T*_*n*_, number of days since sowing.

The total germination percentage was calculated at the end of the 21 days. Three‐day intervals were selected to reduce data point congestion and improve the clarity and readability of the line graphs. The total number of leaves was recorded. Thereafter, the culm and root lengths of seedlings were measured with a digital vernier caliper (*n* = 25 × 5). Seedlings were then dried in an oven (Afrimart, South Africa) at 55°C for 3 days, and total biomass was determined using a precision balance (SigmaAldrich, USA). The seedling vigor index (SVI) was calculated as follows: SVI = seedling length (cm) × germination percentage (Orchard 1977).

In another set of experiments, seeds of *P. australis* and *P. mauritianus* were moistened with the above‐mentioned seawater concentrations and exposed to 5°C/5°C and 35°C/40°C thermoperiods (8‐h night and 16‐h day) for 21 days. These extreme temperatures were chosen because *P. australis* populations in the Midlands are exposed to frost during winter and high temperatures in summer. None of these seeds germinated under those conditions. After 21 days, these ungerminated seeds were transferred to growth chambers and exposed to a constant temperature of 25°C (8‐h night, 16‐h day) at their respective treatments in Petri dishes. Germinated seed counts and germination recovery percentages were determined after 21 days. Seeds that failed to germinate after 21 days were considered non‐viable, as this period is generally sufficient for germination, and seed dormancy was not assessed in this study.

### 2.2 Data Analyses

All data were first screened for normality using the Shapiro–Wilk test. Where data remained non‐parametric, inter‐treatment differences were determined by the Kruskal‐Wallis *post hoc* test. Percentage data were log‐transformed before parametric analyses. Inter‐treatment differences in germination capacity and speed, culm height, leaf number, root length, SVI, and biomass were tested using analysis of variance (ANOVA, SPSS Version 29). Multiple mean comparisons were made using Tukey's *post hoc* test. The repeated‐measures ANOVA was employed to test the effects of salinity, species, and their interactions on germination. Pearson's correlation coefficient was used to test for relationships between salinity and selected variables. All differences were considered significant at *p* < 0.05.

## 3 Results

### 3.1 Control Treatments

**FIGURE 1:**
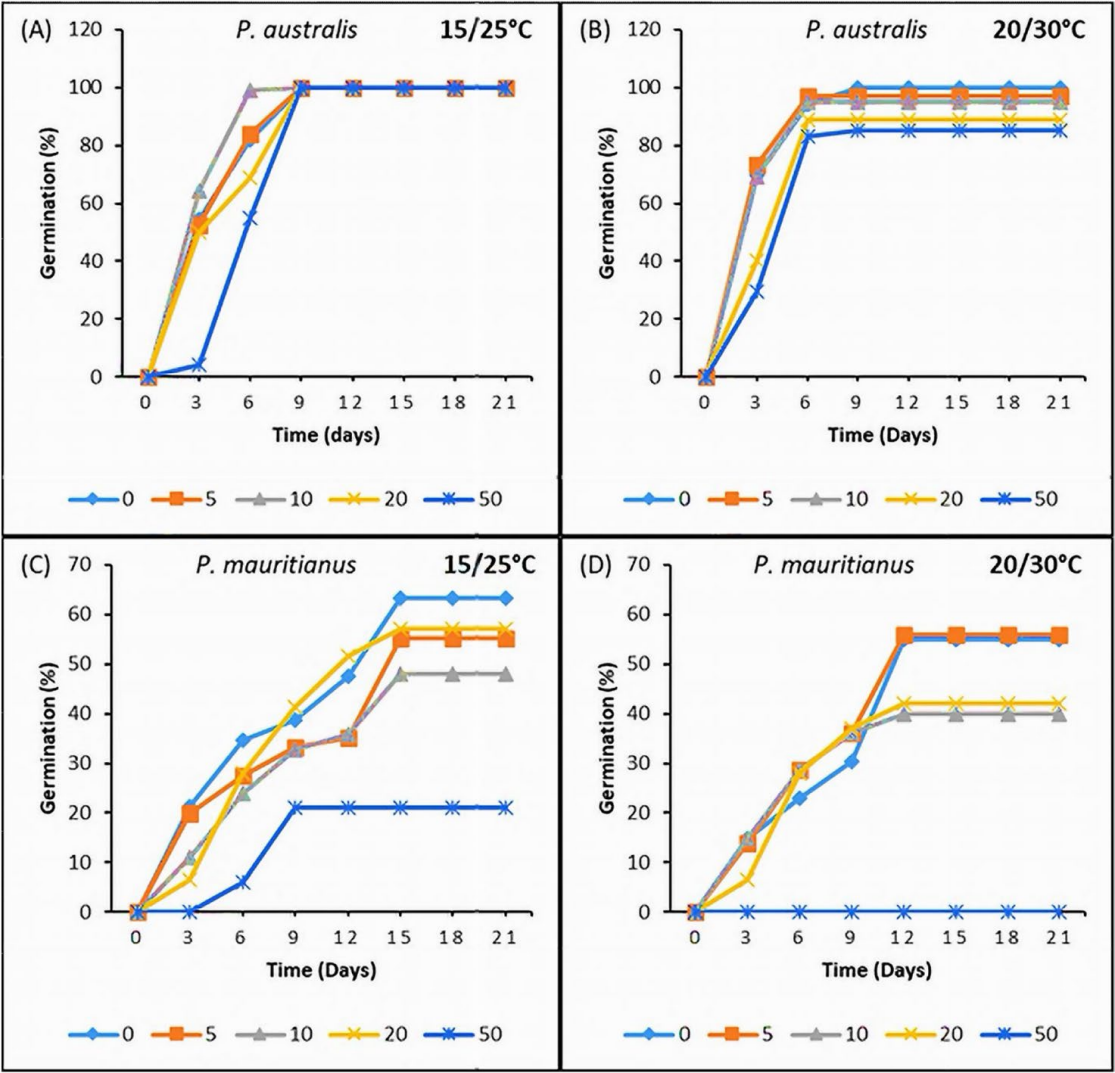
Effects of salinity on seed germination of *P. australis* (A, B) and *P. mauritianus* (C, D) at 3‐day intervals for 21 days. Seeds were subjected to 15°C/25°C (left) or 20°C/30°C (right) thermoperiods (8 h night, 16 h day). Different colors of the legends represent salinity concentrations. Mean ± SE are indicated, *n* = 125; Tukey's post hoc test (*p* < 0.05). SE are too small to be visible.

**FIGURE 2:**
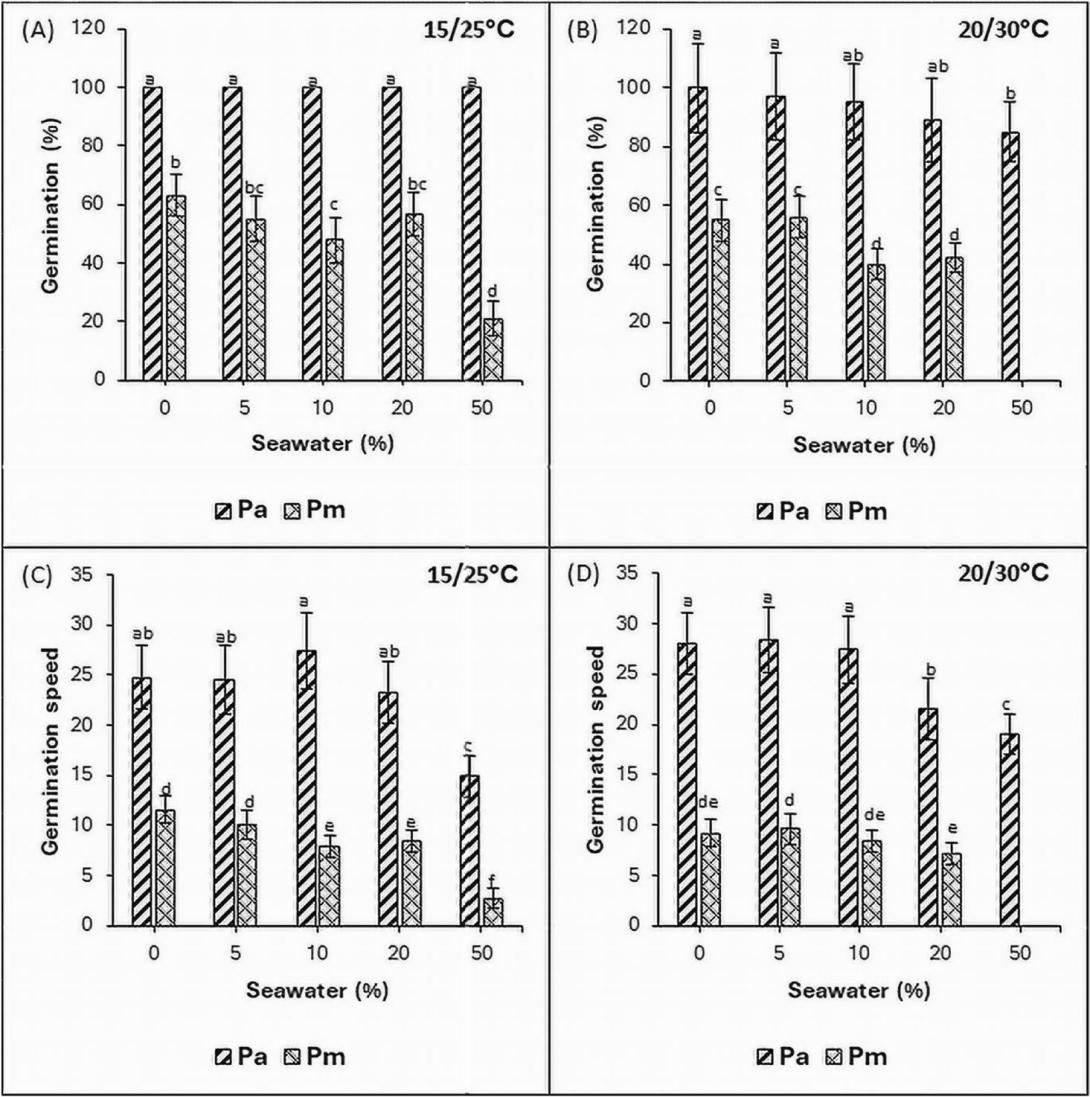
Effects of salinity on seed germination (A, B) and germination speed (C, D) of *P. australis* (Pa) and *P. mauritianus* (Pm) after 21 days of exposure to 15/25°C (left) and 20/30°C (right) thermoperiods (8 h night, 16 h day) at different salinities. Mean ± SE are indicated, *n* = 125; Tukey's post hoc test (*p* ≤ 0.05), bars with different letters are significantly different.

In the non‐saline control treatments, there was 100% germination in *P. australis* at 15°C/25°C and 20°C/30°C thermoperiods, while in *P. mauritianus*, germination was 36% and 45% lower than control (*p* < 0.05), respectively (Figures 1 and 2). Seedlings of both species germinated better at a thermoperiod of 15°C/25°C compared to 20°C/30°C. Moreover, *P. australis* attained 50% germination in the non‐saline control treatment within 3 days compared to 13 days in *P. mauritianus* (Table 1). The rate of germination was significantly greater in *P. australis* than in *P. mauritianus* (Figure 2).

**TABLE 1:** Time taken to reach 50% germination in *P. australis* and *P. mauritianus* . Seeds were exposed to 0%, 5%, 10%, 20%, and 50% seawater at 15°C/25°C and 20°/30° thermoperiods (8 h night, 16 h day) for 21 days. Values represent means (*n* = 125).

Treatments (% seawater)	Days to reach 50% germination			
	15°C/25°C		20°C/30°C	
	*P. australis*	*P. mauritianus*	*P. australis*	*P. mauritianus*
0	3	13	2	11
5	3	14	2	11
10	3	15	2	40% in 21 days
20	3	12	4	42% in 21 days
50	5	21% < 50%	5	0% in 21 days

### 3.2 Saline Treatments

Seeds of *P. australis* attained 100% germination at all salinities in the 15/25°C thermoperiod, reaching 50% germination within 3 days, except for the 50% seawater treatment, which took 5 days (Figures 1 and 2). In contrast, the germination percentage of *P. mauritianus* was less than 70% across all salinities, being highest in the control and significantly reduced in the salinity treatments. In the 50% seawater treatment, *P. mauritianus* had the lowest germination percentage (21%) of all treatments in the 15°C/25°C thermoperiod. In the 20°C/30°C thermoperiod, seed germination in both species was highest in the control and significantly lower in the salinity treatments. In *P. australis*, germination was significantly reduced in the 20% and 50% seawater treatments compared to the control (*p* < 0.05). In *P. mauritianus* , seeds in the 50% seawater treatment failed to germinate (Table 1).

### 3.3 Leaf Number

**FIGURE 3:**
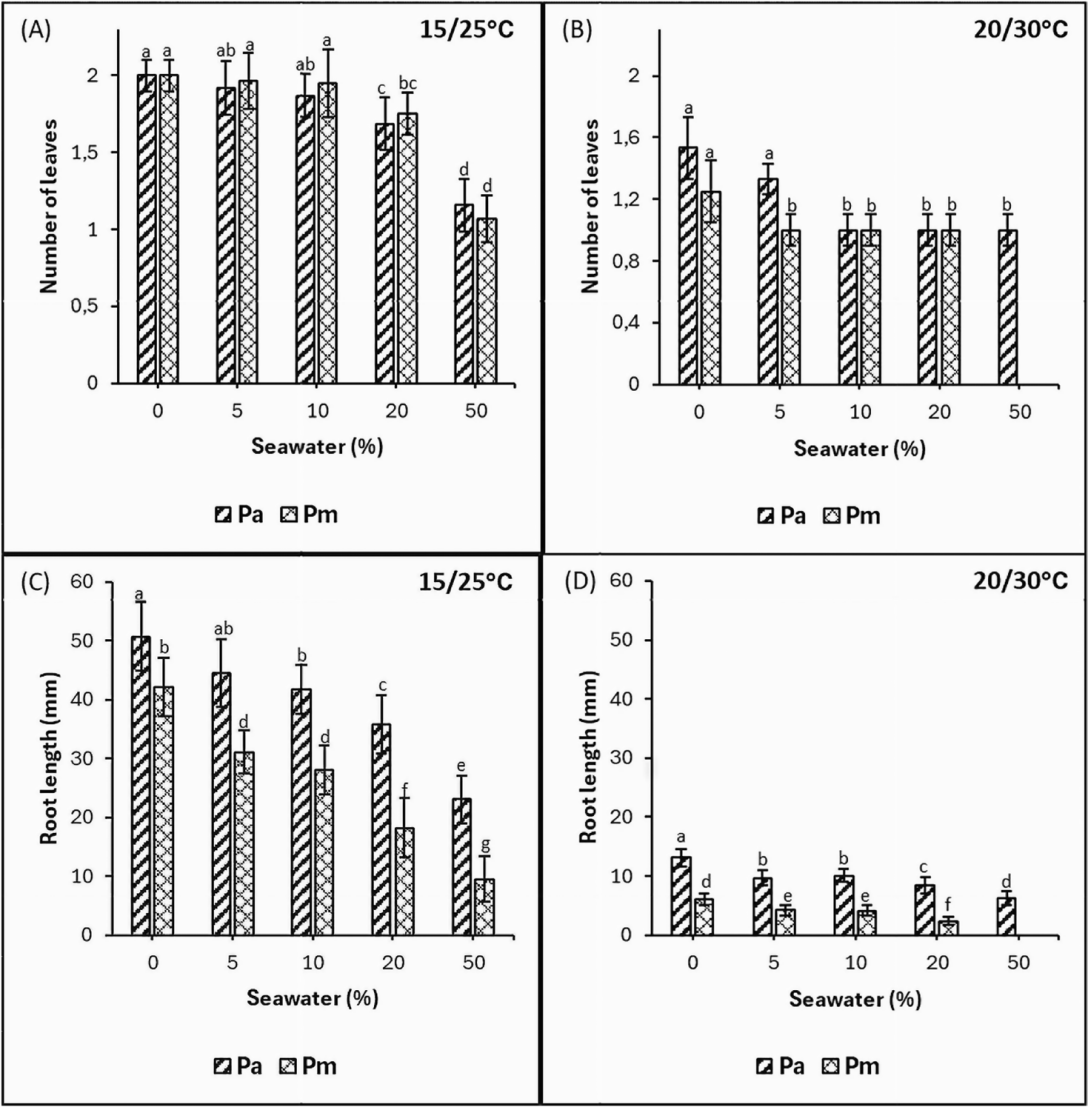
Effects of salinity on the total number of leaves (A, B) and root length (C, D) of *P. australis* (Pa) and *P. mauritianus* (Pm) seedlings after 21 days exposure to 15°C/25°C (left) and 20°C/30°C (right) thermoperiods (8 h night, 16 h day) at different salinities. Means ± SE are indicated, *n* = 125; Tukey's post hoc test (*p* ≤ 0.05), bars with different letters are significantly different.

In the 15°C/25°C thermoperiod, both species produced significantly greater numbers of leaves in the 0%, 5%, and 10% compared to the 20% and 50% seawater treatments (*p* < 0.05) (Figure 3). The 50% seawater treatment significantly reduced the number of leaves in both species compared to higher salinities. There were no differences in leaf number between species in their response to salinity in the 15°C/25°C thermoperiod, while in the 20°C/30°C thermoperiod, an increase in salinity significantly reduced the number of leaves in both species. In this thermoperiod, *P. australis* produced significantly more leaves in the 0% and 5% than at the higher salinities, while in *P. mauritianus* , the leaf number was highest in the control and significantly lower in the saline treatments (*p* < 0.05).

### 3.4 Root Length

In both thermoperiods, root length was greater in *P. australis* than in *P. mauritianus* and decreased with an increase in salinity (Figure 3). In *P. mauritianus* , there was a significant negative relationship between the root length and salinity in both thermoperiods (*R* ^2^: 0.884 and *R* ^2^: 0.784, Table 3).

### 3.5 Culm Height

**FIGURE 4:**
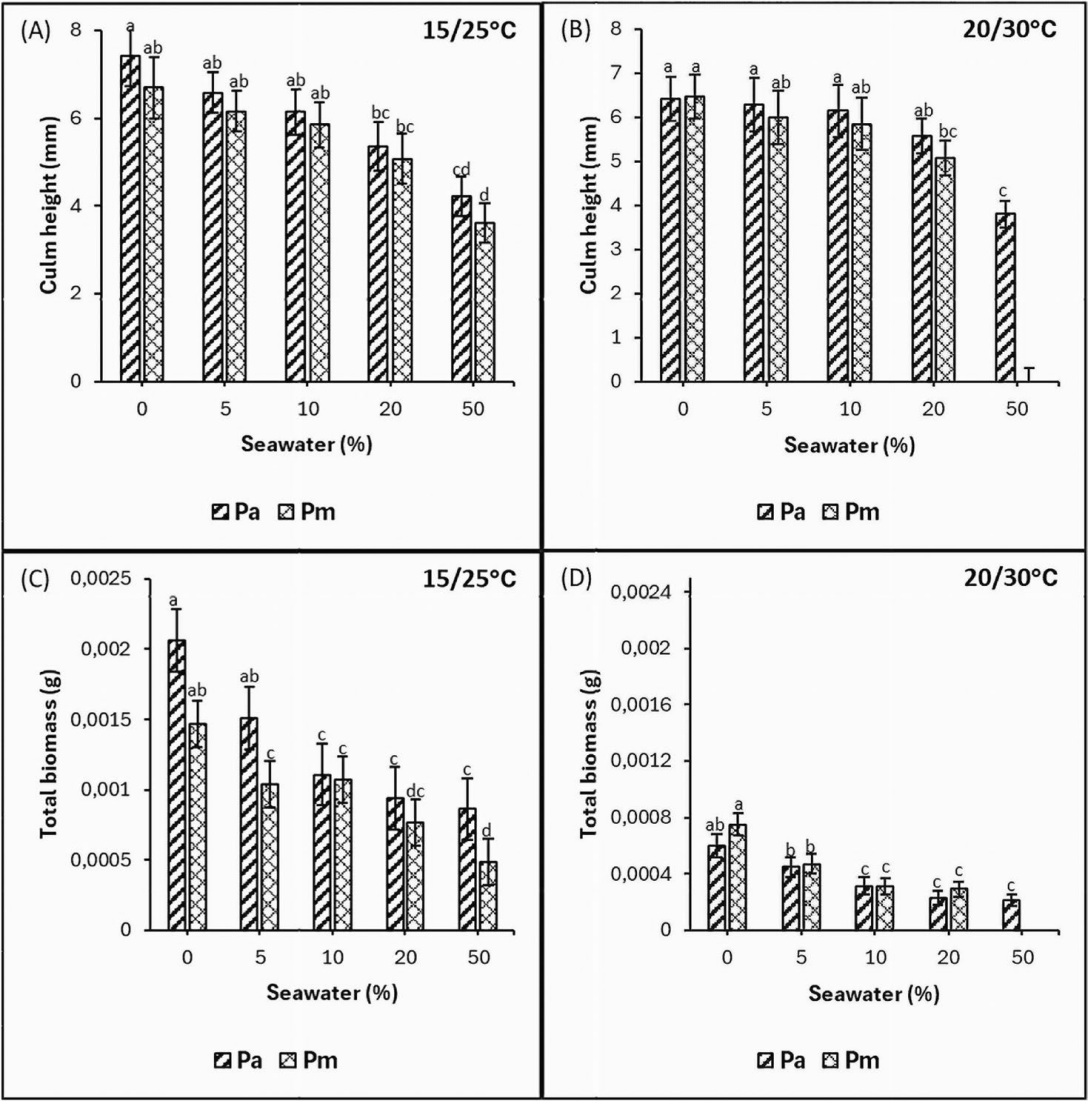
Effects of salinity on culm height (A, B) and total biomass (C, D) of *P. australis* (Pa) and *P. mauritianus* (Pm) seedlings after 21 days exposure to 15°C/25°C (left) and 20°C/30°C (right) thermoperiods (8 h night, 16 h day) at different salinities. Mean ± SE are indicated, *n* = 125; Tukey's post hoc test (*p* ≤ 0.05), bars with different letters are significantly different.

Culm height in both species decreased with an increase in salinity in both thermoperiods (*p* < 0.05) and was significantly lower under the 50% seawater treatment (Figure 4). The culm height was highest in the control compared to higher salinities in both thermoperiods (*p* < 0.05).

### 3.6 Seedling Biomass

Seedling biomass was highest in the control and decreased with an increase in salinity in both thermoperiods (*p* < 0.05). At the 15°C/25°C thermoperiod, *P. australis* had greater biomass than *P. mauritianus* at 5% and 50% seawater treatments (Figure 4). In both species, biomass was greater in the 15°C/25°C than in the 20°C/30°C thermoperiod. At the latter temperature regime, biomass declined with increasing salinity, indicating a negative correlation between salinity and biomass accumulation (Table 3).

### 3.7 Seedling Vigor Index (SVI)

**FIGURE 5:**
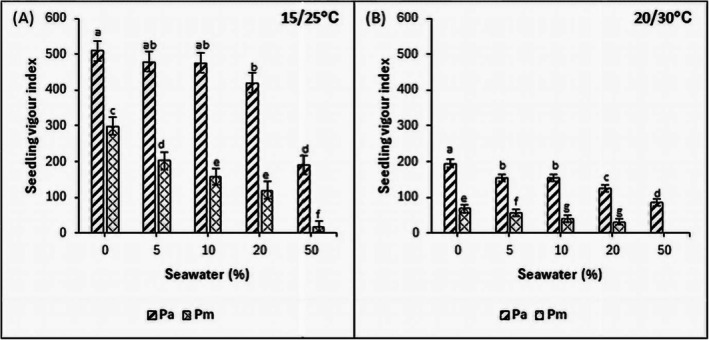
Effects of salinity on seedling vigor index of *P. australis* (Pa) and *P. mauritianus* (Pm) seedlings after 21 days exposure to 15°C/25°C (A) and 20°C/30°C (B) thermoperiods (16 h day, 8 h night) at different salinities. Values represent mean ± SD (*n* = 125); Tukey's post hoc test (*p* ≤ 0.05), bars with different letters are significantly different.

The SVI in both species was highest in the control and decreased with increasing salinity in both thermoperiods (*p* < 0.05). The SVI of *P. australis* was significantly higher than that of *P. mauritianus* in both thermoperiods (Figure 5). *Phragmites mauritianus* exhibited a strong negative correlation between SVI and salinity at 25°C/15°C (R^2^: 0.789) and 30°C/25°C (R^2^: 0.774) thermoperiods, indicating that seedling vigor declined with increasing salinity (Table 3).

### 3.8 Germination at Extreme Temperatures

In both species, there was no germination in the 5°C/5°C and 35°C/40°C thermoperiods after 21 days. After transfer of the ungerminated seeds to 25°C, *P. australis* exhibited germination recovery of 80% and 47% compared to 16% and 0%, respectively, in *P. mauritianus* (Table 2).

**TABLE 2:** The germination recovery percentage of *P. australis* and *P. mauritianus* after being exposed to 5/5°C and 35/40° thermoperiods, (8 h night, 16 h day) for 21 days. Seeds were exposed to 0%, 5%, 10%, 20% and 50% seawater. Seeds were exposed to 25°C, and recovery % was determined. Values represent mean ± SE (*n* = 125), Tukey's post hoc test (*p* ≤ 0.05), for the mean difference.

Treatments (% seawater)	Gemination recovery (%)			
	5/5°C		35/40°C	
	*P. australis*	*P. mauritianus*	*P. australis*	*P. mauritianus*
0	80 ± 0.93^a^	16 ± 1.03^ab^	47 ± 0.79^ab^	0^a^
5	44 ± 0.81^c^	20 ± 0.99^a^	57 ± 0.77^a^	0^a^
10	48 ± 0.52^c^	12 ± 1.09^bc^	58 ± 0.56^a^	0^a^
20	68 ± 0.47^b^	24 ± 0.89^a^	48 ± 0.44^ab^	0^a^
50	32 ± 0.64^d^	0^d^	04 ± 0.25^c^	0^a^
*p*	0.01	0.01	0.02	0.29
*p* ≤ 0.05				

^a,b,c,d^Within a column, means without a common superscript differ (*p* ≤ 0.05).

**TABLE 3:** Pearson's correlation coefficients in *P. australis* and *mauritianus* seedlings to salinity for various variables (*p* ≤ 0.05). Bold indicates strong relationships between variables.

Variable	15°C/25°C		20°C/30°C	
	*P. australis*	*P. mauritianus*	*P. australis*	*P. mauritianus*
Number leaves	*R* ^2^: 0.609	*R* ^2^: 0.677	*R* ^2^: 0.523	*R* ^2^: 0.500
Root length	***R*** ^**2**^ **: 0**.**706 *p* < 0,001**	***R*** ^**2**^ **: 0**.**884 *p* < 0,001**	***R*** ^**2**^ **: 0.707 *p* < 0,001**	***R*** ^**2**^ **: 0.784 *p* < 0,001**
Culm height	*R* ^2^: 0.607	*R* ^2^: 0.601	*R* ^2^: 0.558	*R* ^2^: 0.650
Biomass	*R* ^2^: 0.540	***R*** ^**2**^ **: 0**.**714 *p* < 0,001**	***R*** ^**2**^ **: 0.770 *p* < 0,001**	***R*** ^**2**^ **: 0**.**874 *p* < 0,001**
Seedling vigor index	*R* ^2^: 0.612	***R*** ^**2**^ **: 0**.**789 *p* < 0,001**	***R*** ^**2**^ **: 0.739 *p* < 0,001**	***R*** ^**2**^ **: 0.774 *p* < 0,001**

*Note:* *p* ≤ 0.05.

### 3.9 Seed Morphology

**FIGURE 6:**
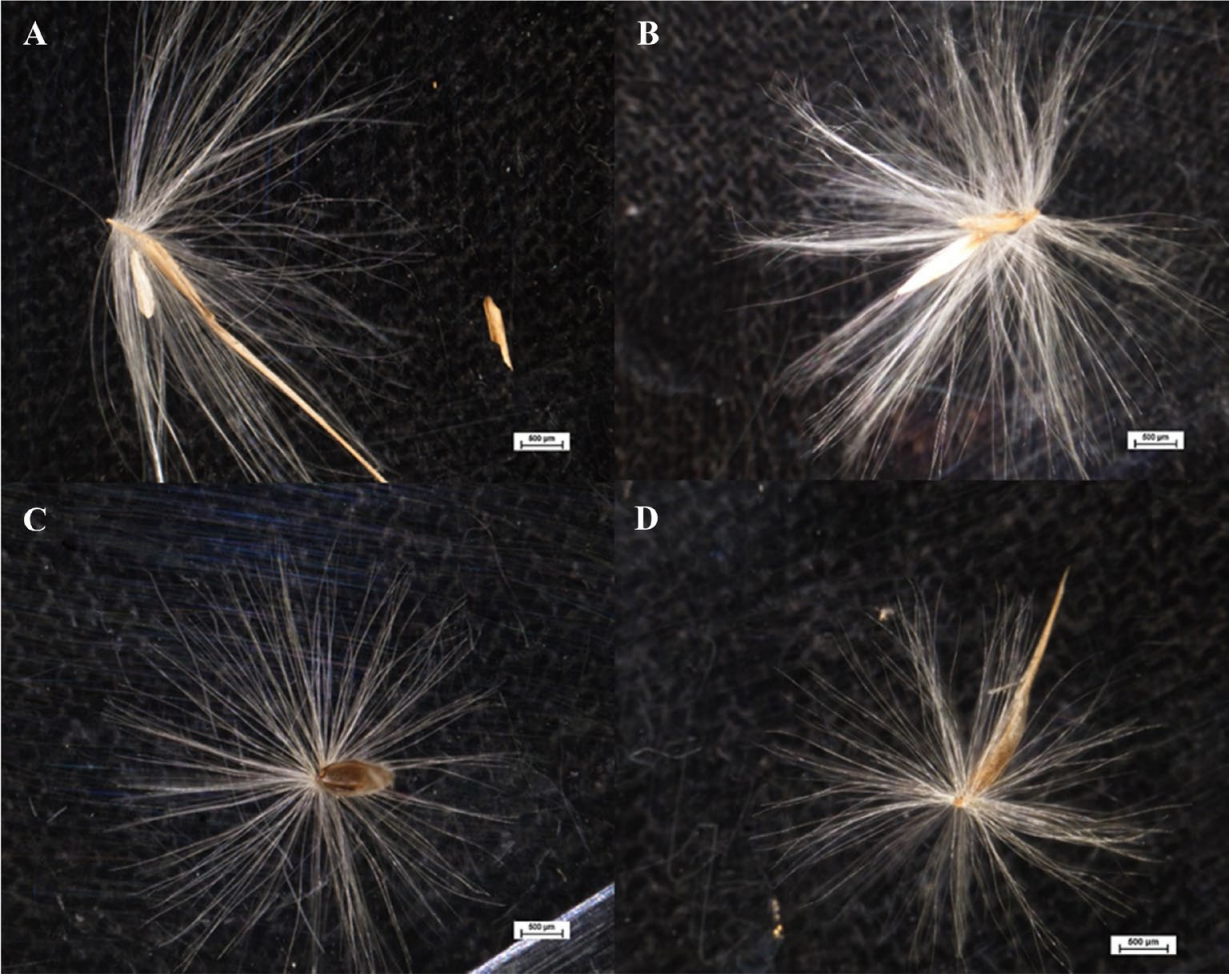
Images of mature seeds of *P. australis* (A, B) and *P. mauritianus* (C, D) using a Nikon Eclipse AZ100 microscope.

Although there were no statistical differences in seed weight between species (S2), *P. australis* seeds were more elongated, hairier, and fluffier than those of *P. mauritianus* (Figure 6).

## 4 Discussion

Seed germination is crucial for the establishment and spread of reed populations. The spread of common reeds can be attributed to their low habitat requirements, efficient dissemination, and vigorous vegetative growth (Brisson et al. 2010; Kettenring et al. 2015). Understanding germination in relation to temperature, salinity, light, and soil moisture is essential for predicting plant colonization and invasion (Baskin and Baskin 2022; Hovick et al. 2023). Temperature and salinity are particularly vital for germination success as they influence the geographic and ecological distribution of *Phragmites* ecotypes (Greenwood and MacFarlane 2006; Chen et al. 2024). Numerous studies have been undertaken on *Phragmites* species worldwide, including *P. australis*, the most common reed (Kettenring et al. 2011; Yu et al. 2012), *P. karka* (Zehra and Khan 2007), and *P. communis* (Harris and Marshall 1960; Gorai et al. 2006). Our study is the first on seed germination on the African haplotypes, *P. australis* and *P. mauritianus*. The results of this research on two coexisting *Phragmites* species may be used to predict their dispersal niches, distribution patterns, and competitive invasion patterns in southern Africa.

In our collection sites, *P. australis* and *P. mauritianus* produced masses of seeds, the vast majority of which were viable soon after collection under favorable conditions. This observation is consistent with findings from a previous study on *P. australis* (Baldwin et al. 2010). Seed viability did not vary significantly between sites within each species; however, it differed between species in response to salinity and temperature tolerance. These results reflect real‐world conditions, suggesting that both species possess the ability to recover from frost, indicating that low temperatures may be less damaging to seed embryos than high temperatures. Some studies reported that seed dormancy is important for the germination of *P. australis* following dispersal and that seeds require cold, moist conditions over the winter to break dormancy in the following spring (Kettenring et al. 2015). In our study and European *P. australis* (Kentnesse 2017), however, seeds were non‐dormant at maturity and germinated immediately after dispersal. Under non‐saline treatments at 15°C/25°C and 20°C/30°C thermoperiods, there was 100% germination in *P. australis*, while in *P. mauritianus*, germination was 36% and 45% lower, respectively. Moreover, *P. australis* attained 50% germination within 3 days compared to 13 days in *P. mauritianus* . These data clearly show that in *P. mauritianus* , seed germination decreased as thermoperiod increased and that *P. australis* seeds germinated more rapidly than *P. mauritianus* at both photoperiods. This study also reveals the low germination capacity of *P. mauritianus* under optimal temperature conditions, due to specific environmental requirements, whereas *P. australis* exhibited a broad tolerance to varying environmental conditions. Others reported that temperatures between 10°C and 30°C were favorable for the germination of *P. australis* (Menge et al. 2016; Ruiz‐Talonia et al. 2019). Optimal seed germination in reed species requires diurnal temperature fluctuations between 10°C and 30°C to stimulate germination (Hazelton et al. 2014). These thermal fluctuations probably provide seeds with a season‐sensing mechanism which delays the onset of germination during autumn and winter until large diurnal temperature fluctuations occur. In addition, the specific temperature requirements probably restrict germination to conditions suitable for seedling establishment in wetlands (Baskin and Baskin 2022). The requirement for large amplitudes may also serve to inhibit germination during submergence or burial in soil (Ungar 2017).

In our study, there were distinct differences in germination between species after exposure to extreme thermoperiods (5°/5° and 35°C/40°C) for 21 days, which were selected to represent the temperature conditions experienced by *P. australis* populations in the Midlands, KwaZulu‐Natal. Seeds of *P. australis* retained greater viability than *P. mauritianus* (80% and 47% compared to 16% and 0%, respectively). Both species possess the ability to recover from frost, indicating that low temperatures may be less damaging to seed embryos than high temperatures. However, due to the specific environmental requirements of *P. mauritianus* , no recovery was observed at 35°C/40°C. This may partly explain the broader global distribution of *P. australis* compared to *P. mauritianus* . This variation among species is likely driven by genetic mutations or adaptations that influence seed metabolism, water uptake, and resistance to aging (Waterworth et al. 2010). Additionally, while some species produce antimicrobial compounds to protect their seeds, others may lack such defenses, making them more vulnerable to fungal infections that compromise viability (Waterworth et al. 2024). In *P. communis*, there was no germination below 6.7°C, while high temperatures inhibited germination (Gorai et al. 2006; Jordanovska and Miskoska‐Milevska 2018). In *P. karka*, there was greater inhibition of germination at 25°C/35°C than at lower temperatures (Zehra et al. 2012). In *P. australis* , germination was low at a constant temperature of 30°C (Greenwood and MacFarlane 2006; Menge et al. 2016). These data indicate that seeds become dormant at extreme temperatures. Dormancy at extreme temperatures is probably an adaptive mechanism to control germination timing, allowing viable dormant seeds to accumulate for future colonization when conditions become favorable (Kettenring and Whigham 2009; Klupczynska and Pawłowski 2021). The seeds of *P. australis* exhibited a higher recovery percentage and greater tolerance to salinity compared to *P. mauritianus* . Studies suggest that seeds under hypersaline conditions may remain viable without germinating, and the ability to remain viable and dormant during unfavorable conditions likely benefits the establishment and dominance of *P. australis* (Greenwood and MacFarlane 2006; Liu et al. 2021).

Salinity plays a critical role in the germination and establishment of plants and is one of the most important factors in determining reed seed germination (Yu et al. 2012). In our study, seed germination of *P. australis* was more rapid than *P. mauritianus* at all salinities in both thermoperiods. In the higher thermoperiod, however, germination of *P. australis* was reduced in the 20% and 50% seawater treatments, while in *P. mauritianus* , germination decreased as salinity increased in both thermoperiods. Research shows that *P. australis* germinates more rapidly under fluctuating thermal conditions, with optimal germination occurring at temperature amplitudes above 10°C. While direct comparisons with *P. mauritianus* are limited, available data suggest that *P. australis* has adaptive traits that enhance its ability to germinate across a wide range of salinity levels and temperature fluctuations (Greenwood and MacFarlane 2006; Bansal et al. 2019). The combination of salinity and thermoperiod significantly reduced biomass, number of leaves, culm height, root elongation, and SVI of both species in both thermoperiods, with effects being greater in *P. mauritianus* compared to *P. australis*. In *P. karka* , salinity inhibited germination more at higher (25°C/35°C) than lower temperatures (Zehra et al. 2012). Others also reported that in *P. australis* (Yu et al. 2021) and *P. communis* (Gorai et al. 2006), the percentage of germination and germination speed decreased with increasing salinities. In our study, germination of *P. australis* was inhibited in 50% seawater, while in both *P. karka* and *P. communis*, some seeds germinated at 400 mM NaCl at temperatures between 20°C and 30°C (Gorai et al. 2006; Zehra et al. 2012), but as temperatures increased, seeds became dormant or lost viability (Zehra and Khan 2007; Baskin and Baskin 2020). Reduced germination at high temperatures or hypersaline conditions is probably an adaptive survival strategy. Our results on germination of *P. australis* are higher than those recorded in North America (Wijte and Gallagher 1996) and Australia (Harris and Marshall 1960) but similar to those reported in China (Yu et al. 2012) and Europe (Mauchamp and Mesleard 2001).

The biomass, root length, and SVI decreased with an increase in salinity in both species. Similarly, elevated salinity has been linked to reduced plant vigor, leading to decreased biomass and root development in *P. australis* (Eller et al. 2017; Guan et al. 2023). Under hypersaline conditions, germination is inhibited by either decreased water absorption or ion toxicity (Ungar 2017). Seeds that do not germinate under high salinities constitute a persistent seed bank, enabling the species to spread germination over the year. In our study, the highest percentage and speed of germination in *P. australis* were achieved in the 5% seawater treatment, suggesting that low salinities may stimulate germination, as reported previously (Yu et al. 2012; Liu et al. 2021). This stimulation probably confers a competitive advantage for *P. australis* over other species under saline conditions when receiving freshwater inputs (Yu et al. 2012). The germination percentage, biomass, root length, and SVI were consistently higher in *P. australis* compared to *P. mauritianus* in all salinities and both thermoperiods. Seed viability, vigor, germination, and dormancy can vary based on genetic diversity and geographical and ecological distribution (Baskin and Baskin 2022). The results of our study indicate that greater plasticity in response to thermoperiod and salinity in *P. australis* may be attributed to genotypic and phenotypic variation between the two species (Mozdzer and Zieman 2010; Eller and Brix 2012). The lower germination and reduced vigor of *P. mauritianus* seeds under increased salinity and temperature are evident in the field, significantly limiting their spread across diverse habitats compared to *P. australis* . The delayed germination of *P. mauritianus* seeds may lead to seed degradation, reduced vigor, and reduced ability of this species to compete with *P. australis* .

The longer, more hairy and fluffy seeds of *P. australis* appear to be lighter, more easily airborne, and dispersed further than those of *P. mauritianus* . The low viability and dispersal capacity of *P. mauritianus* seeds significantly limit their ability to occupy broad habitats. Studies have shown that *Typha latifolia* relies on wind pollination and the production of large quantities of small seeds. However, dormancy and a low survival percentage often hampered their germination due to specific environmental requirements (Meng et al. 2016). In conclusion, these findings support our hypothesis and highlight clear ecological differences between *P. mauritianus* and *P. australis* , with one species exhibiting more invasive characteristics than the other. *Phragmites mauritianus* is less salt‐tolerant, less invasive, and generally absent from hypersaline estuaries, likely due to its specific environmental requirements. It also displays low seed viability, limited genetic and phenotypic diversity, and a sparse distribution, all of which constrain its ability to establish and persist across varied habitats. In contrast, *P. australis* is a cosmopolitan species that thrives across a wide range of environmental conditions, including saline wetlands (Pompeiano et al. 2017). It employs both sexual and asexual reproductive strategies and has a broad ecological amplitude, enabling it to colonize and dominate diverse wetland ecosystems (Mal and Narine 2004; Hurley et al. 2024). Overall, the study highlights that species with low seed viability, reduced genetic diversity, and narrow ecological tolerance, such as *P. mauritianus* , are more vulnerable to environmental pressures, particularly under the increasing impacts of climate change.

## 5 Conclusion

In both species, there was greater germination and seedling vigor under non‐saline conditions, which was consistently higher in *P. australis* than in *P. mauritianus* . Germination percentage, time taken to reach 50% germination, and seedling vigor were significantly higher in *P. australis* than in *P. mauritianus* at all salinities and in both thermoperiods, probably due to greater genetic diversity. The combination of salinity and elevated temperature exacerbated stress, significantly reducing biomass, leaf production, culm height, and root elongation in seedlings of both species. This suggests that global warming and sea‐level rise, as consequences of climate change, could severely hinder the growth of common reeds, particularly in coastal and low‐lying ecosystems. No germination was observed at 5°C or under 35°C/40°C thermoperiods in both species; however, *P. australis* seeds demonstrated higher viability, as evidenced by greater germination recovery percentages compared to *P. mauritianus* . Phenotypic qualities of *P. australis*, such as lighter and more hairy seeds, may also contribute to greater dispersal and the ability to colonize more diverse habitats than *P. mauritianus* . Consequently, the low viability, plasticity, and limited dispersal capacity of *P. mauritianus* seeds could significantly restrict their ability to occupy diverse habitats; therefore, lower government expenditure on control efforts. The high seed viability and survival rate of the salt‐tolerant exotic *P. australis* , coupled with its aggressive asexual reproduction, pose significant concerns for environmental managers. In contrast, *Phragmites mauritianus* is highly sensitive to salinity, reducing its likelihood of spreading toward estuaries. Additionally, its low seed viability prevents it from dominating natural ecosystems, thereby reducing concerns for environmental managers. These findings align with recent observations showing *P. australis* advancing toward river mouths, where it threatens fish migration and recreational activities. Meanwhile, no evidence of *P. mauritianus* has been recorded in hypersaline coastal environments. This research provides robust and reliable insights that can aid environmental managers in effectively addressing the challenges posed by the invasive *Phragmites* species.

## Conflicts of Interest

The authors declare no conflicts of interest.

## Supporting information


**Data S1:** pei370091‐sup‐0001‐Supinfo.docx.

## Data Availability

The data supporting the findings of this study are available in the [raw data for germination] at [https://zenodo.org/records/16926464/files/raw%20data%20for%20germination.xlsx?download=1&preview=1].
