# Supplementary material for: Seed Germination in Phragmites australis and P. mauritianus: Effects of Salinity and Thermoperiod
Source: Plant Environ Interact. 2025 Oct 6;6(5):e70091. doi: 10.1002/pei3.70091 (PMC12500532; doi:10.1002/pei3.70091)
Supplement: Supplementary file 1 — Data S1: pei370091‐sup‐0001‐Supinfo.docx. [file PEI3-6-e70091-s001.docx]

**Supplementary data**

S1/Table 1: Effects of salinity, species and their interaction in a Two factorial ANOVA for various variables (P ≤ 0.05).

| **25°C** | | | **30°C** | | |
| --- | --- | --- | --- | --- | --- |
| **Root length** | *F* | *P* | *F* | | *P* |
| Salinity | 146,476 | <0.001 | 103,529 | | <0.001 |
| Species | 280,542 | <0.001 | 868,667 | | <0.001 |
| Salinity * Species | 2,559 | <0.05 | 1,573 | | >0.01 |
| **Biomass** | | | | | |
| Salinity | 7,432 | <0.001 | 73,650 | | <0.001 |
| Species | 6,521 | <0.05 | 0,014 | | >0.01 |
| Salinity * Species | 0,530 | >0.01 | 7,650 | | <0.001 |
| **Number of leaves** | | | | | |
| Salinity | 161,24 | <0.001 | | 51,708 | <0.001 |
| Species | 0,576 | >0.01 | | 103,547 | <0.001 |
| Salinity * Species | 0,112 | >0.01 | | 25,953 | <0.001 |
| **Germination %** |  | | | | |
| Salinity | 44,914 | <0.001 | | 9,348 | <0.001 |
| Species | 721,514 | <0.001 | | 319,94 | <0.001 |
| Salinity * Species | 44,914 | <0.001 | | 4,250 | <0.05 |
| **Culm height** |  | | | | |
| Salinity | 15,92 | <0.001 | | 23,071 | <0.001 |
| Species | 2,717 | >0.01 | | 0,656 | >0.01 |
| Salinity * Species | 0,278 | >0.01 | | 0,679 | <0.05 |

**Root Length**

Salinity had a significant effect on root length at both 25 °C and 30 °C. The two species responded differently to salinity stress, showing significant interspecific variation in root length. At 25 °C, salinity significantly affected root length across species, whereas at 30 °C, the effect of salinity and its interaction with species was minimal.

**Biomass**

Salinity had a strong effect on the biomass of both species at both temperatures. At 25°C, significant differences in biomass in responses to salinity across species. In contrast, no significant differences in biomass between species at 30°C. there was no interacting with between salinity and species responses at 25°C however was not at 30°C.

**Number of Leaves**

Salinity had a significant effect on the total number of leaves at both temperatures (p < 0.001). At 25°C, there were no significant interspecific differences in leaf number in response to salinity. However, at 30°C, a strong and significant difference was observed between species. This indicates that both species responded similarly to salinity at 25°C, but differently at 30°C.

**Germination Percentage**

Salinity had a significant effect on germination percentage in both species. The species responded differently to salinity under both thermoperiods, resulting in significant interspecific differences in response. The interaction effects between salinity and species were significant at both temperatures, with a stronger effect observed at 25°C.

**Culm Height**

Salinity had a significant effect on culm height in both species. There were no interspecific differences in response at both thermoperiods. The interaction between salinity and species was not significant at 25°C (p > 0.01), indicating that both species responded similarly to salinity under this thermoperiod. However, at 30°C, the interaction was marginally significant (p < 0.05), suggesting that the species began to diverge in their germination responses to salinity under higher temperatures.

S2/Figure 1: Seeds weight (150) of both species collected from the field. Values represent mean±SD (n=5). Values with different letters are significantly different, independent t test (p > 0.05).
